# Safety Monitoring of Bivalent COVID-19 mRNA Vaccine Booster Doses Among Children Aged 5–11 Years — United States, October 12–January 1, 2023

**DOI:** 10.15585/mmwr.mm7202a5

**Published:** 2023-01-13

**Authors:** Anne M. Hause, Paige Marquez, Bicheng Zhang, John R. Su, Tanya R. Myers, Julianne Gee, Sarada S. Panchanathan, Deborah Thompson, Tom T. Shimabukuro, David K. Shay

**Affiliations:** ^1^CDC COVID-19 Emergency Response Team; ^2^Food and Drug Administration, Silver Spring, Maryland.

On October 12, 2022, the Food and Drug Administration (FDA) issued Emergency Use Authorizations (EUAs) for bivalent (mRNA encoding the spike protein from the SARS-CoV-2 ancestral strain and BA.4/BA.5 Omicron variants) formulations of Pfizer-BioNTech and Moderna mRNA COVID-19 vaccines for use as a single booster dose ≥2 months after completion of primary series or monovalent booster vaccination for children aged 5–11 years (Pfizer-BioNTech) and 6–17 years (Moderna); on December 8, 2022, FDA amended the EUAs to include children aged ≥6 months (*1*,*2*). The Advisory Committee on Immunization Practices (ACIP) recommends that all persons aged ≥6 months receive an age-appropriate bivalent mRNA booster dose (*3*). The safety of bivalent mRNA booster doses among persons aged ≥12 years has previously been described (*4*). To characterize the safety of bivalent mRNA booster doses among children aged 5–11 years after receipt of bivalent Pfizer-BioNTech and Moderna booster doses, CDC reviewed adverse events and health impacts reported to v-safe,* a voluntary, smartphone-based U.S. safety surveillance system established by CDC to monitor adverse events after COVID-19 vaccination, and to the Vaccine Adverse Event Reporting System (VAERS), a U.S. passive vaccine safety surveillance system co-managed by CDC and FDA^†^ (*5*). During October 12–January 1, 2023, a total of 861,251 children aged 5–11 years received a bivalent Pfizer-BioNTech booster, and 92,108 children aged 6–11 years received a bivalent Moderna booster.^§^ Among 3,259 children aged 5–11 years registered in v-safe who received a bivalent booster dose, local (68.7%) and systemic reactions (49.5%) were commonly reported in the week after vaccination. Approximately 99.8% of reports to VAERS for children aged 5–11 years after bivalent booster vaccination were nonserious. There were no reports of myocarditis or death after bivalent booster vaccination. Eighty-four percent of VAERS reports were related to vaccination errors, 90.5% of which did not list an adverse health event. Local and systemic reactions reported after receipt of a bivalent booster dose are consistent with those reported after a monovalent booster dose; serious adverse events are rare. Vaccine providers should provide this information when counseling parents or guardians about bivalent booster vaccination. Preliminary safety findings from the first 11 weeks of bivalent booster vaccination among children aged 5–11 years are reassuring. Compared with the low risk of serious health effects after mRNA COVID-19 vaccination, the health effects of SARS-CoV-2 infection include death and serious long-term sequalae (*6*). ACIP recommends that all persons aged ≥6 months receive an age-appropriate bivalent mRNA booster dose ≥2 months after completion of a COVID-19 primary series or receipt of a monovalent booster dose.^¶^

A parent or guardian with a v-safe account can register a child aged <15 years and complete health surveys on behalf of the child.** Health surveys sent daily during the week after vaccination ask questions about local injection site and systemic reactions and health impacts experienced; registrants can provide additional information about these reactions or health impacts via free text responses. CDC’s v-safe call center personnel contact registrants who report receiving medical care to request further information; registrants are also encouraged to complete a VAERS report, if indicated.

VAERS accepts reports of postvaccination adverse events from health care providers, vaccine manufacturers, and members of the public.^††^ Providers in the CDC COVID-19 Vaccination Program are required to file VAERS reports for observed adverse events after vaccination and for vaccination errors. Signs, symptoms, and diagnoses reported to VAERS are assigned Medical Dictionary for Regulatory Activities preferred terms (MedDRA PTs) by VAERS personnel.^§§^ Death certificates and autopsy reports are requested for any reported death.

A bivalent booster dose in v-safe was defined as administration of an age-appropriate mRNA COVID-19 vaccine dose on or after October 12, 2022, to a registrant who had completed at least a primary vaccination series (2 doses of Pfizer-BioNTech or Moderna vaccine). In this report, local and systemic reactions and health impacts reported during the week after vaccination were described for v-safe registrants aged 5–11 years who received a bivalent booster dose during October 12–January 1, 2023. VAERS adverse event reports were described by serious and nonserious classification, demographic characteristics, and MedDRA PTs. Reports of serious events to VAERS were reviewed by CDC physicians to form a consensus on clinical impression based on available data.^¶¶^ Possible cases of myocarditis, a rare adverse event that has been associated with mRNA COVID-19 vaccines, were identified using selected MedDRA PTs (*6*). All analyses were conducted using SAS software (version 9.4; SAS Institute). These surveillance activities were reviewed by CDC and conducted consistent with applicable federal law and CDC policy.***

## Review of v-safe Data

During October 12–January 1, 2023, a total of 3,259 v-safe registrants aged 5–11 years received an age-appropriate bivalent booster dose (Table 1); 2,647 (81.2%) received Pfizer-BioNTech, and 612 (18.8%) received Moderna bivalent booster doses. Approximately 20.6% (670) of registrants received at least one other vaccination at the same visit as bivalent booster vaccination; among these, 649 (96.9%) received an influenza vaccine.

**TABLE 1:** Demographic and vaccination characteristics reported to v-safe for children aged 5–11 years* who received a bivalent Pfizer-BioNTech or Moderna COVID-19 vaccine booster dose**^†^** — United States, October 12–January 1, 2023

Characteristic	No. (%), by vaccine		
	Pfizer-BioNTech (n = 2,647)	Moderna (n = 612)	Total (N = 3,259)
Sex			
Female	1,296 (49.0)	300 (49.0)	**1,596 (49.0)**
Male	1,338 (50.6)	310 (50.7)	**1,648 (50.6)**
Unknown	13 (0.5)	2 (0.3)	**15 (0.5)**
**Age range, yrs (median)**	5–11 (8)	6–11 (8)	**5–11 (8)**
**Ethnicity**			
Hispanic or Latino	298 (11.3)	51 (8.3)	**349 (10.7)**
Non-Hispanic or Latino	2,293 (86.6)	549 (89.7)	**2,842 (87.2)**
Unknown	56 (2.1)	12 (2.0)	**68 (2.1)**
**Race**			
American Indian or Alaska Native	7 (0.3)	2 (0.3)	**9 (0.3)**
Asian	131 (5.0)	27 (4.4)	**158 (4.9)**
Black or African American	99 (3.7)	16 (2.6)	**115 (3.5)**
Native Hawaiian or other Pacific Islander	5 (0.2)	0 (—)	**5 (0.2)**
White	2,033 (76.8)	489 (79.9)	**2,522 (77.4)**
Multiracial	245 (9.3)	58 (9.5)	**303 (9.3)**
Other	71 (2.7)	8 (1.3)	**79 (2.4)**
Unknown	56 (2.1)	12 (2.0)	**68 (2.1)**
**Total no. of COVID-19 vaccine doses received**			
3	1,055 (39.9)	119 (19.4)	**1,174 (36.0)**
4	1,588 (60.0)	493 (80.6)	**2,081 (63.9)**
5	4 (0.1)	0 (—)	**4 (0.1)**
**Vaccine co-administration^§^**			
Yes	565 (21.3)	105 (17.2)	**670 (20.6)**
No	2,082 (78.7)	507 (82.8)	**2,589 (79.4)**

* On October 12, 2022, the Food and Drug Administration authorized bivalent (mRNA encoding the spike protein from the SARS-CoV-2 ancestral strain and BA.4/BA.5 Omicron variants formulations of Pfizer-BioNTech and Moderna mRNA COVID-19 vaccines for use as a single booster dose ≥2 months after completion of primary series or monovalent booster vaccination for children aged 5–11 years and 6–17 years, respectively. A bivalent booster dose in v-safe was defined as an age-appropriate mRNA vaccine dose administered on or after October 12, 2022, for registrants who had completed at least a primary series (2 doses of Pfizer-BioNTech or Moderna vaccine).

^†^ Includes registrants who completed at least one survey during postvaccination days 0–7.

^§^ Other vaccines administered during the same visit.

On ≥1 day during the week after receipt of the bivalent booster dose, local injection site reactions were reported for 1,740 (65.7%) Pfizer-BioNTech recipients and 470 (76.8%) Moderna recipients (Table 2); systemic reactions were reported for 1,215 (45.9%) Pfizer-BioNTech recipients and 379 (61.9%) Moderna recipients. The most commonly reported adverse reactions after receipt of either vaccine were injection site pain (2,146; 65.9%), fatigue (1,076; 33.0%), and headache (745; 22.9%). Most reported reactions were mild in severity (noticeable, but not problematic). Reactions were most frequently reported the day after vaccination; reporting frequency decreased in the days that followed. At least 1 day during the week after bivalent booster vaccination, 469 (14.4%) children were reported to be unable to attend school, and 447 (13.7%) were unable to complete daily activities. Sixty-two (1.9%) parents or guardians reported seeking medical care for their child after bivalent booster vaccination, most commonly in an outpatient clinic (37; 1.1%); no children received hospital care. Of the 64 reports of medical care sought, 37 had additional information available; parents or guardians of 35 children reported that seeking care was unrelated to vaccination.

**TABLE 2:** Adverse reactions and health impacts reported to v-safe for children aged 5–11 years* who received a bivalent Pfizer-BioNTech or Moderna COVID-19 vaccine booster dose, by vaccine — United States, October 12–January 1, 2023

Event^†^	No. (%) reporting reaction or health impact after vaccination^§^		
	Pfizer-BioNTech (n = 2,647)	Moderna (n = 612)	Total (N = 3,259)
**Any injection site reaction**	1,740 (65.7)	470 (76.8)	**2,210 (67.8)**
Pain	1,683 (63.6)	463 (75.7)	**2,146 (65.9)**
Swelling or hardness	229 (8.7)	64 (10.5)	**293 (9.0)**
Redness	211 (8.0)	64 (10.5)	**275 (8.4)**
Itching	123 (4.7)	21 (3.4)	**144 (4.4)**
**Any systemic reaction**	1,215 (45.9)	379 (61.9)	**1,594 (48.9)**
Fatigue	798 (30.2)	278 (45.4)	**1,076 (33.0)**
Headache	534 (20.2)	211 (34.5)	**745 (22.9)**
Fever	512 (19.3)	198 (32.4)	**710 (21.8)**
Myalgia	353 (13.3)	145 (23.7)	**498 (15.3)**
Chills	247 (9.3)	103 (16.8)	**350 (10.7)**
Nausea	208 (7.9)	89 (14.5)	**297 (9.1)**
Abdominal pain	182 (6.9)	56 (9.2)	**238 (7.3)**
Vomiting	115 (4.3)	39 (6.4)	**154 (4.7)**
Joint pain	106 (4.0)	41 (6.7)	**147 (4.5)**
Diarrhea	74 (2.8)	15 (2.5)	**89 (2.7)**
Rash	37 (1.4)	8 (1.3)	**45 (1.4)**
**Any health impact**	506 (19.1)	196 (32.0)	**702 (21.5)**
Unable to attend school	355 (13.4)	114 (18.6)	**469 (14.4)**
Unable to perform normal daily activities	298 (11.3)	149 (24.4)	**447 (13.7)**
Needed medical care	49 (1.9)	13 (2.1)	**62 (13.9)**
Outpatient clinic	30 (1.1)	7 (1.1)	**37 (1.1)**
Telehealth	10 (0.4)	4 (0.7)	**14 (0.4)**
Other	12 (0.5)	3 (0.5)	**15 (0.5)**
Emergency department visit	4 (0.1)	0 (—)	**4 (0.1)**
Hospitalization	0 (—)	0 (—)	**0 (—)**

* On October 12, 2022, the Food and Drug Administration authorized bivalent (mRNA encoding the spike protein from the SARS-CoV-2 ancestral strain and BA.4/BA.5 Omicron variants) formulations of Pfizer-BioNTech and Moderna mRNA COVID-19 vaccines for use as a single booster dose ≥2 months after completion of primary series or monovalent booster vaccination for children aged 5–11 years and 6–17 years, respectively. A bivalent booster dose in v-safe was defined as an age-appropriate mRNA vaccine dose administered on or after October 12, 2022, for registrants who had completed at least a primary series (2 doses of Pfizer-BioNTech or Moderna vaccine).

^†^ Events reported are not mutually exclusive.

^§^ Percentage of registrants reported a reaction or health impact at least once during postvaccination days 0–7.

## Review of VAERS Data

During October 12–January 1, 2023, VAERS received and processed 922 reports of adverse events among children aged 5–11 years (Table 3).^†††^ The median recipient age was 9 years (range = 5–11 years), and 459 (49.8%) reports were for females. Approximately 13.4% (124) of registrants received at least one other vaccination during that same visit; of those, 115 (92.7%) received an influenza vaccine. Among all 922 VAERS reports, 920 (99.8%) were classified as nonserious, 845 (99.8%) after Pfizer-BioNTech and 75 (100%) after Moderna bivalent booster vaccination.

**TABLE 3:** Events* reported to the Vaccine Adverse Event Reporting System for children aged 5–11 years**^†^** after receipt of a bivalent Pfizer-BioNTech or Moderna COVID-19 vaccine booster dose — United States, October 12–November 20, 2022

Adverse events	No. (%) reporting, by vaccine		
	Pfizer-BioNTech (n = 847)	Moderna (n = 75)	Total (N = 922)
**Serious reports^§^**			
**Total serious reports**	**2 (0.2)**	**0 (—)**	**2 (0.2)**
**Nonserious reports**			
**Total nonserious reports**	**845 (99.8)**	**75 (100)**	**920 (99.8)**
**Reports of vaccination error** ^¶^	726 (85.9)	49 (65.3)	**775 (84.2)**
Error without adverse health event	661 (91.0)	40 (81.6)	**701 (90.5)**
Error with adverse health event**	65 (9.0)	9 (18.4)	**74 (9.5)**
**Reports not specifying vaccination error^††^**	119 (14.1)	26 (34.7)	**145 (15.8)**
Fever	13 (10.9)	8 (30.8)	**21 (14.5)**
Syncope	17 (14.3)	3 (11.5)	**20 (13.8)**
Vomiting	10 (8.4)	8 (30.8)	**18 (12.4)**
Nausea	12 (10.1)	5 (19.2)	**17 (11.7)**
Dizziness	12 (10.1)	2 (7.7)	**14 (9.7)**
Fall	11 (9.2)	1 (3.9)	**12 (8.3)**
Fatigue	6 (5.0)	5 (19.2)	**11 (7.6)**
Headache	5 (4.2)	6 (23.1)	**11 (7.6)**
Loss of consciousness	11 (9.2)	0 (—)	**11 (7.6)**
Cough	7 (5.9)	2 (7.7)	**9 (6.21)**
Urticaria	7 (5.9)	2 (7.7)	**9 (6.21)**

**Abbreviations**: MedDRA PT = Medical Dictionary for Regulatory Activities preferred term; VAERS = Vaccine Adverse Event Reporting System.

* Signs and symptoms in VAERS reports are assigned MedDRA PTs by VAERS staff members. Each VAERS report might be assigned more than one MedDRA PT, which can include normal diagnostic findings. A MedDRA PT does not indicate a medically confirmed diagnosis.

^†^ On October 12, 2022, the Food and Drug Administration authorized bivalent (mRNA encoding the spike protein from the SARS-CoV-2 ancestral strain and BA.4/BA.5 Omicron variants) formulations of Pfizer-BioNTech and Moderna mRNA COVID-19 vaccines for use as a single booster dose ≥2 months after completion of primary series or monovalent booster vaccination for children aged 5–11 years and 6–17 years.

^§^ The most common MedDRA PTs among reports of vaccination error included incorrect dose administered (303; 39.1%), incorrect product formulation administered (207; 26.7%), product preparation issue (177; 22.8%), and product administered to patient of inappropriate age (126; 16.3%).

^¶^ The most common adverse health events MedDRA PTs for reports with nonserious vaccination errors included fever (24; 32.4%), pain in extremity (20; 27.0%), fatigue (14; 18.9%), headache (11; 14.9%), and pain (eight; 10.8%).

** Excluding reports of vaccination error. Includes the top 10 most frequently coded MedDRA PTs among nonserious reports.

^††^ VAERS reports are classified as serious if any of the following are reported: hospitalization, prolongation of hospitalization, life-threatening illness, permanent disability, congenital anomaly or birth defect, or death. Serious reports to VAERS were reviewed by CDC physicians to form a clinical impression. https://www.meddra.org/how-to-use/basics/hierarchy

The most common events reported (775; 84.2%) were vaccination errors (e.g., incorrect dose administered [303; 39.1%], incorrect product formulation administered [207; 26.7%], product preparation issue [177; 22.8%], and product administered to patient of an inappropriate age [126; 16.3%]). Reports assigned the MedDRA PTs “incorrect dose administered,” “incorrect product formulation administered,” or “product administered to patient of inappropriate age” often represented situations in which a child received an adult bivalent booster dosage or a bivalent booster dose instead of the appropriate monovalent primary series dose. Reports assigned the MedDRA PT “product preparation issue” often represented situations in which vaccine was incorrectly reconstituted. Among 775 reports of vaccination errors related to bivalent booster vaccination, 74 (9.5%) reports indicated that an adverse health event had occurred.

After excluding vaccination error reports, 145 (15.8%) of the 920 nonserious reports remained. Commonly reported events included fever (21; 14.5%), syncope (20; 13.8%), vomiting (18; 12.4%), nausea (17; 11.7%), and dizziness (14; 9.7%). Two serious reports were for children who received Pfizer-BioNTech vaccine; one for a child who developed symptoms consistent with Miller Fisher syndrome, a rare, acquired neurologic condition considered to be a variant of Guillain-Barré syndrome^§§§^; verification based on medical record review is pending. The other one was for a child hospitalized with urticaria and arthritis. No reports of myocarditis or death after bivalent booster vaccination were received.

## Discussion

This report provides findings from v-safe and VAERS data collected during the first 11 weeks of bivalent Pfizer-BioNTech and Moderna mRNA booster dose administration among children aged 5–11 years; during this period, approximately 953,359 booster doses were administered to children in this age group. The findings in this report are generally consistent with those from postauthorization vaccine safety surveillance of monovalent mRNA COVID-19 booster vaccination in this age group (*7*).

Reports to v-safe of systemic and injection site reactions after bivalent booster vaccination among children aged 5–11 years were similar in frequency to those reported after monovalent Pfizer-BioNTech booster vaccination (*7*). Consistent with previous descriptions of reactogenicity after mRNA COVID-19 vaccination (*8*), reactions and health impacts were reported more frequently for children who received Moderna than for those who received Pfizer-BioNTech bivalent booster vaccination. Most reports to v-safe of children who received medical care after bivalent booster vaccination indicated that care was not related to vaccination.

After administration of >950,000 doses of bivalent booster vaccine to children aged 5-11 years, only two serious VAERS reports have been received. Approximately 99.8% of reports to VAERS for children aged 5–11 years after bivalent booster vaccination were deemed nonserious; most (85.0%) reports were related to vaccination errors. Many vaccination errors represented children receiving an incorrect bivalent booster dose for their age or an incorrectly reconstituted dose. Most reports of vaccination error did not include an adverse health event; those with an event were consistent with expected reactions after an mRNA COVID-19 vaccination. Among events reported to VAERS, vaccination errors were reported with a similar frequency among children aged 5–11 years after monovalent (71%) or bivalent (84%) booster vaccination (*7*). Vaccination errors represented a smaller proportion of events (35%) reported among persons aged ≥12 years who received bivalent booster vaccination (*4*). CDC provides updated clinical guidance, educational materials, and training opportunities after each update to COVID-19 vaccine recommendations.^¶¶¶^ Public health officials should continue to provide training materials for vaccine administrators to help reduce vaccination errors among children.

The findings in this report are subject to at least four limitations. First, v-safe is a voluntary program, and data might not be representative of the vaccinated population. Second, v-safe does not directly identify whether a vaccine is monovalent or bivalent; therefore, misclassification might occur among children who aged into this population without having completed a 3-dose primary series. Third, VAERS is a passive surveillance system and subject to reporting biases and underreporting, especially of nonserious events (*5*). Finally, conclusions drawn from these data are limited by the 11-week surveillance period; safety monitoring will continue during the bivalent booster vaccination program.

ACIP recommends that all persons aged ≥6 months receive an age-appropriate bivalent mRNA booster dose ≥2 months after completion of a COVID-19 primary series or receipt of a monovalent booster dose. Preliminary safety findings from the first 11 weeks of bivalent booster vaccination among children aged 5–11 years are reassuring. Compared with the low risk of serious health effects after mRNA COVID-19 vaccination, the health effects of SARS-CoV-2 infection include death and serious long-term sequalae (*6*). Immunization with bivalent vaccines provides significant additional protection against symptomatic SARS-CoV-2 infection (*9*). CDC and FDA will continue to monitor vaccine safety and will provide updates as needed to help guide COVID-19 vaccination recommendations.

**Summary**

**What is already known about this topic?**

After CDC’s October 2022 recommendation for bivalent COVID-19 booster vaccination for children aged 5–11 years, children in this age group received approximately 953,359 bivalent booster doses during October 12, 2022–January 1, 2023.

**What is added by this report?**

Early safety findings from v-safe and the Vaccine Adverse Event Reporting System (VAERS) for bivalent booster vaccination in children aged 5–11 years are similar to those described for monovalent booster vaccination. Most VAERS reports represented vaccine errors rather than adverse events. Neither myocarditis nor death were reported after bivalent booster vaccination.

**What are the implications for public health practice?**

These preliminary safety findings should be provided when counseling parents or guardians about bivalent booster vaccination. All eligible persons should receive a bivalent booster dose.

## Footnotes

*
https://vsafe.cdc.gov/en

†
https://vaers.hhs.gov

§ https://covid.cdc.gov/covid-data-tracker/#vaccination-demographic (Accessed January 1, 2023).

¶ https://www.cdc.gov/vaccines/covid-19/clinical-considerations/covid-19-vaccines-us.html(Accessed January 1, 2023).

** Text message reminders are sent to parents or guardians to complete online health surveys for their child on days 0–7 after vaccination; then weekly through 6 weeks after vaccination; and then 3, 6, and 12 months after vaccination. Previously registered persons can report receipt of a COVID-19 booster dose, and new registrants can enter information about all doses received; registrants can also indicate whether any other vaccines were administered during the same visit. Parents and guardians use the following definitions to describe the severity of a child’s symptoms: mild (noticeable, but not problematic), moderate (limit normal daily activities), or severe (make daily activities difficult or impossible).

†† Under EUA, and as enrolled providers in the CDC COVID-19 Vaccination Program, health care providers are required to report certain adverse events after COVID-19 vaccination to VAERS, including death (https://vaers.hhs.gov/faq.html). VAERS forms ask for patient, vaccine, administration, and adverse event information. https://vaers.hhs.gov/docs/VAERS%202.0_Checklist.pdf

§§ Each VAERS report might be assigned more than one MedDRA PT. A MedDRA-coded event does not indicate a medically confirmed diagnosis. https://www.meddra.org/how-to-use/basics/hierarchy

¶¶ VAERS reports are classified as serious (based on FDA C.F.R. Title 21) if any of the following are reported: hospitalization, prolongation of hospitalization, life-threatening illness, permanent disability, congenital anomaly or birth defect, or death. https://www.accessdata.fda.gov/scripts/cdrh/cfdocs/cfcfr/cfrsearch.cfm?fr

*** 45 C.F.R. part 46, 21 C.F.R. part 56; 42 U.S.C. Sect. 241(d); 5 U.S.C. Sect. 552a; 44 U.S.C. Sect. 3501 et seq.

††† Processed VAERS reports are those that have been coded using MedDRA, deduplicated, and undergone standard quality assurance and quality control review.

§§§ https://www.ninds.nih.gov/health-information/disorders/miller-fisher-syndrome#:~:text%20=%20Miller%20Fisher%20syndrome%20is%20a,preceded%20by%20a%20viral%20illness

¶¶¶
https://www.cdc.gov/vaccines/covid-19/index.html
